# Quality improvement strategies to improve inpatient management of small and sick newborns across All Babies Count supported hospitals in rural Rwanda

**DOI:** 10.1186/s12887-021-02544-z

**Published:** 2021-02-19

**Authors:** David Tuyisenge, Samuel Byiringiro, M. Louise Manirakiza, Robert G. Mutsinzi, Alphonse Nshimyiryo, Merab Nyishime, Lisa R. Hirschhorn, Francois Biziyaremye, Joseph Gitera, Kathryn Beck, Catherine M. Kirk

**Affiliations:** 1 Partners In Health/Inshuti Mu Buzima, PO. Box 3432, Kigali, Rwanda; 2 Feinberg School of Medicine, Northwestern University, 625 N Michigan Avenue, 60611 Chicago, IL USA; 3 Rwandan Ministry of Health, KN 3 Rd, Kigali, Rwanda

**Keywords:** Quality improvement, Neonatal care, Neonatology, Learning collaboratives, Quality of care

## Abstract

**Background:**

Neonatal mortality contributes to nearly half of child deaths globally and the majority of these deaths are preventable. Poor quality of care is a major driver of neonatal mortality in low- and middle-income countries. The All Babies Count (ABC) intervention was designed to reduce neonatal mortality through provision of equipment and supplies, training, mentorship, and data-driven quality improvement (QI) with peer-to-peer learning through learning collaborative sessions (LCS). We aim to describe the ABC scale-up in seven rural district hospitals from 2017 to 2019 focusing on the QI strategies implemented in hospital neonatal care units (NCUs) and the resultant neonatal care outcomes.

**Methods:**

A pre-post quasi experimental study was conducted in 7 rural hospitals in Rwanda in two phases. The baseline periods were April-June 2017 for Phase I and July-September 2017 for Phase II; with end-line data collected during the same periods in 2019. Data included facility audits of supplies and staffing, LCS surveys of QI skills, and reports of implemented QI change ideas. Data on NCU admissions and deaths were extracted from Health Management Information System (HMIS). Facility-reported change ideas were coded into common themes. Changes in post-post neonatal mortality were measured using Chi-squared tests.

**Results:**

NCUs were run by a median of 1 nurse [interquartile range (IQR):1–2] at baseline and endline. Median NCU admissions increased from 121 [IQR: 77–155] to 137 [IQR: 79–184]. Availability of advanced equipment improved (syringe pumps: 57–100 %, vital sign monitors: 51–100 % and CPAP machine: 14–100 %). There were significant improvements in QI skills among NCU staff. All 7 NCUs (100 %) addressed non-adherence to protocol as a priority gap, 5 NCUs (86 %) also improved communication with families. NCU case fatality rate declined from 12.4 to 7.8 % (*p* = 0.001).

**Conclusions:**

The ABC package of interventions combining the provision of essential equipment to NCU, clinical training and strong mentorship, QI coaching, and the LCS approach for peer-to-peer learning was associated with significant neonatal mortality reduction and services utilization in the intervention hospitals.

## Background

Globally, four million neonatal deaths are registered annually, representing nearly half of under-five child mortality [1]. 73 % of neonatal deaths occur in the first week of life, especially during the first 24 hours after delivery [1]. 98 % of all neonatal deaths occur in low- and middle-income countries (LMICs), and 77 % of those in Asia and Sub-Saharan Africa [1]. The estimated average neonatal mortality rate in LMICs in 2017 was 26 per 1,000 live births, compared to 3 per 1000 in high-income countries [2], and hence targeting this high-risk group is an urgent policy priority. The main underlying causes of neonatal deaths include complications of prematurity, birth asphyxia, and infections [3] and the majority of them are preventable through evidence-based clinical interventions [4]. A recent report showed that over half of neonatal deaths in LMIC are the result of poor quality of care compared to a lower proportion due to lack of access to healthcare [5]. However, implementing evidence-based interventions in resource-limited settings can be challenging due to health system constraints [6]. The Sustainable Development Goals (SDGs) have set a target to reduce neonatal mortality to 12 per 1,000 live births or less by 2030 [7]. However, the universal achievement of this goal requires the reversal of the tide of poor quality care which contribute to the majority of preventable deaths, particularly among newborns [8]. Scaling up evidence-based interventions for the management of small and sick newborns is one strategy which could reduce neonatal mortality by 30 % globally [3]. This requires the availability of inpatient specialized care for small and sick newborns, ideally in dedicated units with skilled staff, and thus there is significant potential for quality improvement (QI) interventions [9].

Efforts to improve the quality of health services have targeted different aspects of the system, including inputs for health systems strengthening, capacity building, and data-driven QI methods. Data-driven QI focuses on teaching providers to analyze the system and identify areas for improvement using tools such as a Fishbone diagram and “Plan, Do, Study, Act” (PDSA) cycles to test changes through implementation [10]. The approach of Learning Collaboratives to promote peer-to-peer learning and exchange of change ideas [11] has shown gains in LMICs [12] and are well-received by healthcare providers [13].

In Rwanda, despite a rapid decline in under-five mortality, the number of deaths in the neonatal period remains high (20 per 1,000 live births) and contributes to 40 % of all under-five deaths [14]. The establishment of a national neonatal care protocol in Rwanda has been a critical step in ensuring the provision of quality care to newborns, however the remaining challenge is the delivery of these quality-services to every newborn and at all times [15]. Therefore, since 2013, Partners In Health/Inshuti Mu Buzima (PIH/IMB) partnered with the Rwanda Ministry of Health (MOH) to implement the All Babies Count (ABC) intervention with the aim of reducing neonatal mortality by 30 % through accelerating improvements in the quality of maternal and newborn care in Rwanda [16]. This paper describes the scale up of the ABC change package [17] to the neonatal care units (NCUs) of seven rural hospitals of Rwanda between 2017 and 2019 and the resulting neonatal outcomes using the Standard for Quality Improvement Reporting Excellence framework (SQUIRE) [18].

## Methods

### Study setting

**Fig. 1:**
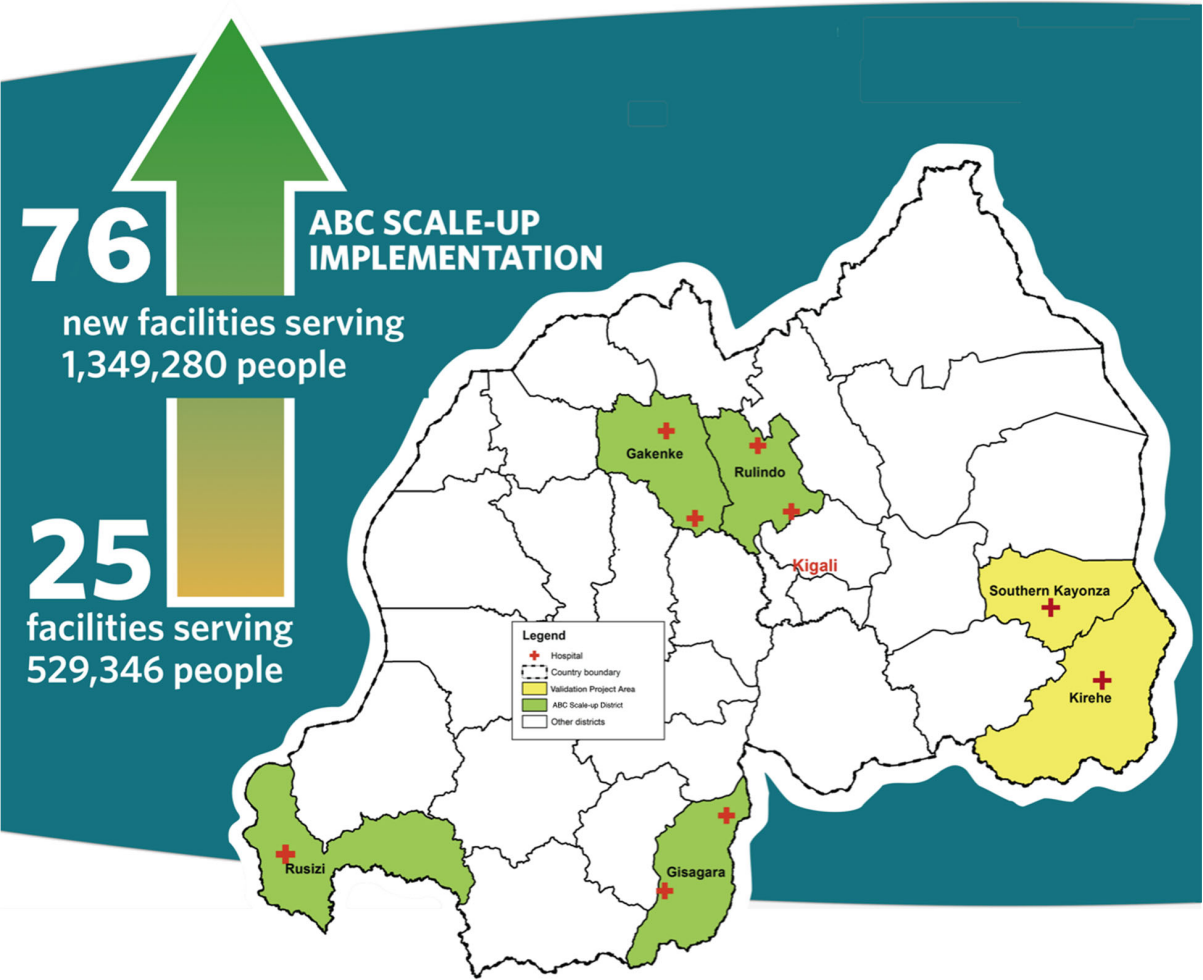
All Babies Count Scale-up Sites

The hospitals for implementation of ABC during scale-up were identified jointly by the Rwandan MOH and PIH/IMB based on the following criteria: high neonatal mortality rate and no partner organizations to improve neonatal care in their areas. The hospitals were Nemba and Ruli in Gakenke District, Kinihira and Rutongo in Rulindo District, Gakoma and Kibilizi in Gisagara District, and Mibilizi in Rusizi District, in the Northern, Southern and Western Provinces of Rwanda respectively (Fig. 1). All hospitals were Rwanda MOH operated facilities and they supervised 69 referring-health centers serving a population of approximately 1,349,280 people. A district-hospital NCU in Rwanda provides phototherapy, oxygen, intravenous fluids, nutrition and breastfeeding, vital signs monitoring, neonatal resuscitation, Kangaroo Mother Care (KMC), and drug administration. The units are led by a nurse in-charge with a supervising medical doctor.

### Intervention

**Fig. 2:**
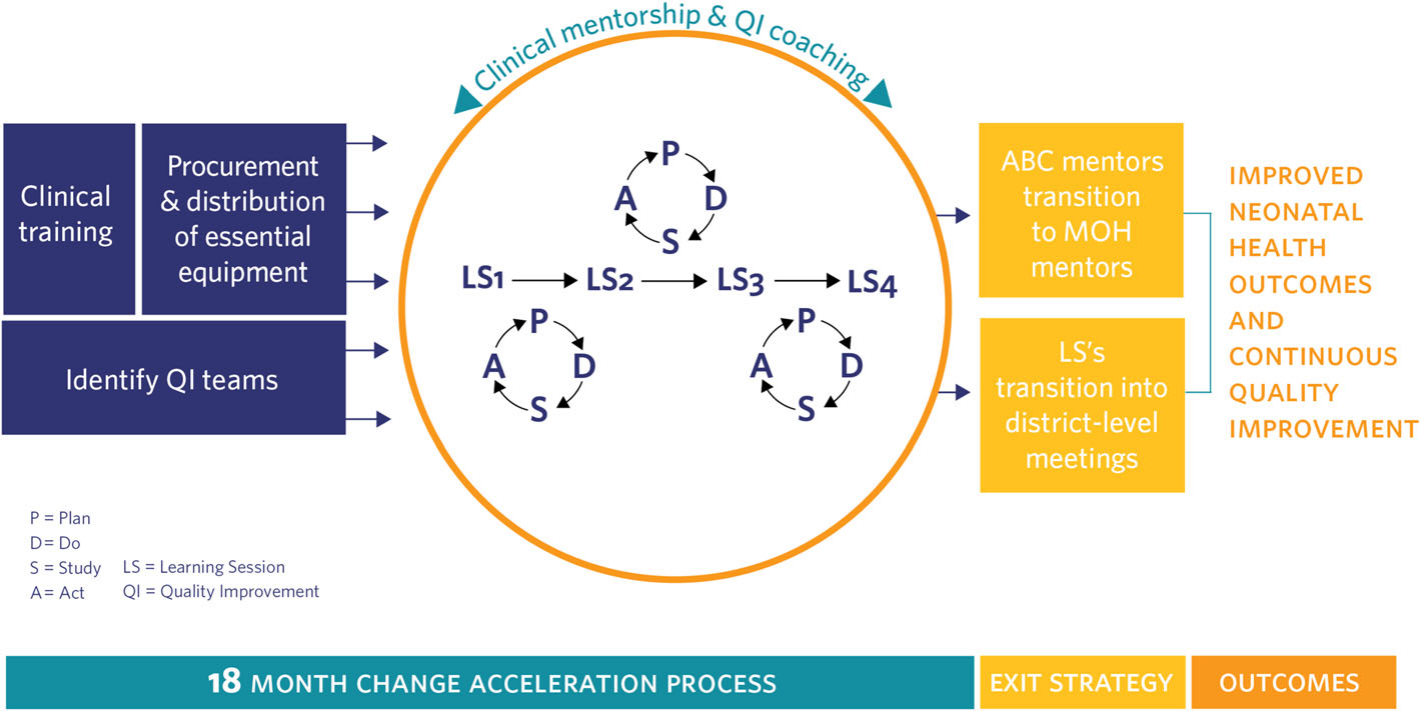
All Babies Count Program Approach

The ABC approach was an 18-month change acceleration process to reduce neonatal mortality through improved quality of care, which is described in depth elsewhere [19]. Before the launch of ABC in each district, a Memorandum of Understanding describing the role of each partner was signed by the MOH, individual participating hospitals, district leadership officials, and PIH/IMB. The ABC intervention package included: (1) clinical training and monthly mentorship, (2) provision of essential equipment and supplies, and (3) a district-wide QI strategy to promote data-use and peer-to-peer learning. A full-time ABC QI Advisor (who was an expert nurse/midwife) was hired as a clinical mentor and QI coach that was responsible for coordinating ABC activities in each participating hospital catchment area. Clinical trainings for neonatal unit staff at the hospital included an on-site five-day training on the national neonatal protocol that was conducted at the very beginning of ABC implementation and a two-day on-site training on Managing Infants with Feeding Difficulties [20] in the NCU to improve breastfeeding that was conducted around the end of the first year of implementation. Trainings included a combination of didactic lectures and hands-on mentored practice during the training using mannequins and direct practice with patients. At the hospital level, trainings targeted nurses, midwives, doctors, and anesthetists who had regular interaction with newborns primarily in the NCU, maternity, and emergency units. The QI Advisor then provided regular mentorship, at least one day per month, for the hospital maternity and NCU as well as all health centers in the hospital catchment area. The package of essential equipment provided was based on a baseline assessment of gaps in standards for newborn care equipment and supplies. Each hospital NCU received equipment based on their needs, most often containing: radiant warmers, Continuous Positive Airway Pressure (CPAP) machines, vital sign monitors, neonatal resuscitation equipment, and electric syringe pumps. Quarterly Learning Collaborative Sessions (LCS) were organized to accelerate uptake of QI through promotion of peer-to-peer learning and data use. The first LCS was organized in the second quarter of ABC intervention, following the initial clinical trainings and mentorship visits. Through the quarterly LCS and monthly QI coaching visits from the QI advisors, facility QI teams learned QI methods to identify gaps in care, experimentation of QI change ideas to improve processes and systems, PDSA cycles, and QI measurement. The LCS were attended by district and health facility leadership, interdisciplinary facility QI teams comprised of nurses/midwives, doctors, supervisors, and monitoring and evaluation staff, and PIH/IMB. In between LCS, facility QI teams were expected to meet monthly. The full model of ABC implementation is described in Fig. 2.

The ABC project instituted multiple strategies to ensure intervention fidelity. ABC QI Advisors submitted a mentor daily log to keep track of mentorship and QI coaching activities. On a monthly basis, the ABC QI Advisor and hospital monitoring and evaluation team made a joint plan of activities which was submitted to the project managers and the hospital leadership. Additionally, there were quarterly check-in meetings with ABC QI Advisors, quarterly reports of ongoing ABC activities to all stakeholders (hospital leadership, district leadership, and the MOH), regular field visits to the ABC sites by a project manager, and quarterly data validation to ensure the quality of data used for evaluation.

### Study Design

The aim of ABC project was to decrease neonatal mortality by 30 % within 18 months of the intervention. This was a pre-post quasi-experimental study conducted in seven rural hospitals that received ABC interventions between 2017 and 2019. Roll-out of ABC occurred in two phases, with Phase I (Gakenke and Rulindo Districts) in June-July 2017 and Phase II (Gisagara and Rusizi Districts) in October-November 2017.

### Data sources

The evaluation of the study included all seven NCUs in the seven public hospitals that received ABC intervention between July 2017 and September 2019. Data were collected through facility audits, mentorship reports, provider surveys, and the national Health Management Information System (HMIS).

Facility audits were conducted by ABC QI Advisors at each hospital to evaluate the number of NCUs staffs and baseline status of essential neonatal care medical equipment. The facility audits were reported in Android tablets using KoboCollect software and conducted prior to the implementation of ABC activities and repeated at the completion of ABC.

The ABC QI Advisor reported (using the KoboCollect software on the Android tablet) all the clinical mentorship and QI coaching visits provided to hospital NCU throughout the course of ABC implementation. These were reported as the average number of mentorship and QI coaching visits reported by ABC QI Advisors to NCUs per month.

We adapted a self-report survey of QI skills and confidence from a Zimbabwe learning collaborative assessment tool [21]. The self-report survey was administered to NCU health care providers at the start of the first and fourth LCS. Participants were asked to rate their own knowledge of different tools used for QI project design and planning (process mapping, root cause analysis, aim setting, prioritization, using PDSA cycles, change ideas development and testing, conducting QI meetings and using QI statistics). Participants rated their knowledge on a Likert scale from 0 (I do not know what this tool is) to 4 (I can teach others this method). At the fourth LCS, we adapted a tool to harvest successful and implemented QI change ideas [17].

The number, reasons, and outcomes of NCU admissions were obtained from HMIS. The data from HMIS were validated through register review by well-trained data officers from PIH/IMB in collaboration with MOH facility teams. HMIS data for Phase I sites were collected from: April – June 2017 (baseline) and April – June 2019 (endline). HMIS data for Phase II sites were collected from: July – September 2017 (baseline) and July – September 2019 (endline).

### Data analysis

We analyzed (1) facility characteristics of NCUs before and after ABC intervention, (2) clinical mentorship and QI coaching in NCUs throughout ABC intervention, (3) QI projects and change ideas tested during ABC intervention, (4) QI skills and confidence among NCU providers, and (5) neonatal unit admissions, morbidity, and mortality. For each hospital, we reported the average number of clinical mentorship and QI coaching visits conducted by the ABC mentor in the NCU per month during the 18-month intervention.

QI project change ideas were coded by the study team into thirteen common themes for areas of improvements and we reported the number and percentages of NCUs that implemented each change idea. In addition, we summarized individual scores obtained by NCU providers on their knowledge and confidence in each QI skill. Finally, we compared the proportion of deaths among all neonatal admissions in all seven NCUs from baseline to endline. We used median and interquartile ranges (IQR) to report continuous variables and frequencies, and percentages for categorical variables. Changes from baseline to endline were assessed using Wilcoxon signed-rank test and Chi-squared tests for continuous and categorical variables, respectively.

### Ethics

The study was approved by the Rwanda National Ethics Committee and the Rwandan Ministry of Health provided approval for access to HMIS data. For individual level data collection during LCS surveys, participants provided written informed consent.

## Results

The median total number of staff assigned to the NCU was four (IQR: 4–5) and five (IQR: 5–5) at the baseline and endline respectively, and a median of one staff was working on day shifts both at baseline and endline (IQR 1–2) (Table 1). All seven hospitals (100 %) had penguin suction bulbs and ambu bags with masks 0 and 1 sizes at both baseline and endline. Six hospitals (85.7 %) had radiant warmers at baseline and five (71.4 %) had them at endline. The hospitals with advanced neonatal equipment increased from baseline to endline: Continuous Positive Airway Pressure (CPAP) devices (14.3 % to 100 %), vital signs monitors (57.1 % to 100 %), electric syringe pumps (57.1 % to 100 %). The availability of essential medicines were as follows at baseline and endline, respectively: aminophylline (85.7 % and 100 %), phenobarbital (85.7 % and 85.7 %), both ampicillin and cefotaxime (100 % and 100 %), gentamycin (85.6 % and 100 %). The main reasons for admission included prematurity and/or low birthweight (LBW) (*n* = 283, 33.3 %) and (*n* = 343, 32.7 %), asphyxia (*n* = 175, 20.6 %) and (*n* = 183,17.4 %) at baseline and endline respectively (Table 1).

**Table 1:** Characteristics of NCUs that received ABC intervention, N = 7 hospitals

	Baseline, n (%)	Endline, n (%)
**Staff**		
Total number of NCU staff, median [IQR]	4 [4-5]	5 [5-5]
Average number of staff on day duty, median [IQR]	1 [1-2]	1 [1-2]
**Availability of functioning essential medical equipment in 7 NCUs**		
Ambu bag for infants with masks Size 0,1	7 (100.0)	7 (100.0)
Infant radiant warmer with Bed	6 (85.7)	5 (71.4)
Tubing for syringe pumps	2 (28.6)	2 (28.6)
Electric syringe pumps	4 (57.1)	7 (100.0)
50 ml syringes for syringe pumps	2 (28.6)	2 (28.6)
Penguin Suction Bulb	7 (100.0)	7 (100.0)
Electric Suction Machine/Aspirator	6 (85.7)	4 (57.1)
Vital Signs Monitors (at least cardio and O2 Sat)	4 (57.1)	7 (100.0)
CPAP	1 (14.3)	7 (100.0)
**Availability of essential medicines in NCUs**		
Aminophylline	6 (85.7)	7 (100.0)
Phenobarbital	6 (85.7)	6 (85.7)
Ampicillin	7 (100.0)	7 (100.0)
Gentamycin	6 (85.7)	7 (100.0)
Cefotaxime (in pharmacy)	7 (100.0)	7 (100.0)
**Main Reasons for NCU admission**		
Total number of admissions in NCU	850 (100.0)	1049 (100.0)
Birth Asphyxia	175 (20.6)	183 (17.4)
Prematurity/LBW	283 (33.3)	343 (32.7)

A median of 20 NCU, maternity and emergency staff (IQR: 20-22.5) were trained per hospital on the National Neonatal Protocol by ABC and 2 (IQR: 2-8.5) were trained on managing Infants with Feeding Difficulties [22] (Table 2). The median number of ABC clinical mentorship visits per month were 3 (IQR: 2–6) and 3 (IQR: 1–4) QI coaching visits during the ABC intervention period.

**Table 2:** Training, clinical mentorship and QI coaching and medical equipment and supplies distribution in the NCU during ABC intervention

	n	%
**Training**		
Staff trained on advanced neonatal care, median [IQR]	20	[20-22.5]
Staff trained on working with infants with feeding difficulties, median [IQR]	2	[2-8.5]
**Clinical mentorship and QI coaching in NCU**		
Number of ABC visits for clinical mentorship per month, Median [IQR]	3	[2-6]
Number of ABC visits for QI coaching per month, Median [IQR]	3	[1-4]

The most commonly implemented change ideas aimed at improving adherence to neonatal care protocol (100 % of hospitals), which included change ideas such as: regular vital signs monitoring (71.4 % of hospitals), and monitoring and adjusting neonatal feeds (42.9 %) (Table 3). In addition, 85.7 % of hospitals implemented change ideas to improve communication with families of patients, which was primarily providing health education and counselling (57.1 % of hospitals). Four hospitals (57.1 %) worked on their own teamwork approach. Three hospitals (42.9 %) promoted family engagement in care. Two hospitals (28.6 %) worked each on availability of equipment and supplies, monitoring processes of care, changes in staffing, infection prevention and control processes, and infrastructure improvements.

**Table 3:** QI projects and tested change ideas during ABC Intervention, N = 7 hospitals

Area of improvement	n (%)	Change ideas tested	n	%
Medical equipment and supplies	2 (28.6 %)	Prevent stockout of medicines (such as aminophylline)	1	14.3 %
		Avail sheets for covering babies	1	14.3 %
		Monitor emergency ambulance kit (medicines) and ensure its completeness for newborns and maternity cases	1	14.3 %
		Avail Personal Protective Equipment (shoes and gowns) for infection prevention and control in NCU	1	14.3 %
Capacity building for NCU staff	5 (85.7 %)	Training of NCU staff on clinical care in neonatal units according to NCU protocol	5	71.4 %
		Training staff on how to use equipment in the NCU	2	28.6 %
		Training of staff on infection prevention and controll for neonatal units	1	14.3 %
		Training on managing infants with feeding difficulties	2	28.6 %
		Sensitize on close monitoring of newborns	1	14.3 %
		Improve monitoring in NCU	1	14.3 %
Documentation	1 (14.3 %)	Proper documentation of fluids electrolytes and nutritional adjustments by medical doctors and nurses	1	14.3 %
Caregiver Engagement in Newborn Care	3 (42.9 %)	Involve caregivers in care using danger signs tracking sheet	1	14.3 %
		Use an “Expert Mom” role model to provide support to other mothers for breastfeeding, KMC, nad observing for danger signs	1	14.3 %
		Task shifting to mothers for hypothermia monitoring by hand with alerts to nurses for temperature taking	1	14.3 %
		Task shifting to mothers for ensuring on-time feeding newborns in NCU after 3 days support by NCU staff	1	14.3 %
Caregiver/client Social Support	1 (14.3 %)	Provide porridge to moms in the NCU	1	14.3 %
Behavior Change Communication (BCC)	5 (85.7 %)	Health education to mothers of babies admitted in NCU on nutrition, breastfeeding, feeding, prevention of hypothermia, danger signs, KMC, hygiene, etc.	4	57.1 %
		Visual reminders on clocks/watches for feeding newborns	1	14.3 %
Process monitoring	2 (28.6 %)	Routine check of vital sign completion	1	14.3 %
		Conduct preterm death audit	1	14.3 %
Protocol Adherence	7 (100 %)	Regular monitoring of vital signs several times per day	5	71.4 %
		Adherence to protocol with regards to neonatal feeding and nutritional adjustment	3	42.9 %
		Daily accurate weight measurement	2	28.6 %
		Check-up for glycemia and temperature (for preterm babies) within 30 min of admission to the NCU	1	14.3 %
		Regular monitoring of newborn weight gain	1	14.3 %
		Monitoring of feeding practices	2	28.6 %
		Change IV lines every 3 days	1	14.3 %
Staffing	2 (28.6 %)	Have the same medical doctor rounding in NCU for 3 consecutive days per week	2	28.6 %
		Increase staff assigned to work in NCU	1	14.3 %
		Appoint permanent staff to NCU (Separating NCU from Maternity)	1	14.3 %
Interfacility Communication	1 (14.3 %)	Health Center communicates the case to hospital NCU by phone call prior to reference of a neonate	1	14.3 %
Teamwork	4 (57.1 %)	QI Team meeting to track the progress of the project	4	57.1 %
		Joint meeting with maternity staff to discuss care of preterm newborns	2	28.6 %
Infection Prevention and Control	2 (28.6 %)	Improve handwashing	1	14.3 %
		Use of alcohol while on ward rounds for everyone	1	14.3 %
		Regular staff deep cleaning of the NCU through “Umuganda”	1	14.3 %
		Hygiene of incubators	1	14.3 %
Infrastructure Improvement	2 (28.6 %)	Relocating NCU to larger existing spaces for improved flow of care	2	29 %

QI skills showed significant improvement across all areas of QI: the use of root cause analysis (fishbone tool) improved from a median of 0 (IQR:0–3) to a median of 3 (IQR:3–4, *p* < 0.001) from LCS one and LCS four respectively (Table 4); using PDSA cycles increased from median of 1 (IQR: 0–3) to median of 3 (IQR: 3–3, *p* = 0.001); and developing, testing and implementing change ideas increased from a median of 2 (IQR 0.5–2.5) to median of 3 (IQR:3–3). The median score on the confidence in using QI methods remained the same from the first LCS with a median of 4 (IQR: 3–5) to the fourth LCS with a median of 4 (IQR:4–5, *p* = 0.1395).

**Table 4:** Self-Reported Assessment of QI Skills and Confidence among NCU care providers at Learning Collaborative Sessions

	LCS 1 Pre-Assess			LCS 4 Pre-Assess			*P*-Value
	**n**	**Median**	**IQR**	**n**	**Median**	**IQR**	
**Quality Improvement Skills**							
Process Mapping	18	0	0, 2	22	3	2, 3	< 0.001
Root Cause Analysis	21	0	0, 3	23	3	3, 4	< 0.001
Aim Setting	21	2	1, 3	23	4	3, 4	< 0.001
Prioritization	21	2	0, 3	22	3	3, 4	< 0.001
Using PDSA Cycles	22	1	0, 3	22	3	3, 3	0.001
Developing, testing and implementing Change Ideas	20	2	0.5, 2.5	22	3	3, 3	0.001
Conducting QI Meetings	21	2	2, 3	22	3	3, 4	0.010
Using QI Statistics	21	2	0, 2	22	3	3, 3	< 0.001
**Confidence in Quality Improvement**	21	4	3, 5	23	4	4, 5	0.1395

The median number of neonatal admissions per NCU was 121 (IQR: 77–155) at baseline and 137 (IQR: 79–184) at endline (*p* = 0.499) (Table 5). In addition, the median number of neonatal admissions per NCU for prematurity and/or LBW was 49 (IQR: 12–58) at baseline and 41 (IQR: 32–55) at endline (*p* = 0.866). The overall case fatality in all seven NCUs declined from 12.4 % at baseline to 7.8 % at endline (*p* = 0.001). The case-fatality rate among preterm and/or LBW neonates in all seven NCUs declined from 18.4 % at baseline to 11.7 % at endline (*p* = 0.018).

**Table 5:** Admissions and mortality in the NCUs before and after ABC

	Baseline		Endline		*P*-Value
	**n**	**%**	**n**	**%**	
Neonatal admissions per NCU, median [IQR]	121	[77–155]	137	[79–184]	0.499
Neonatal deaths per NCU, median [IQR]	13	[11-22]	12	[6-17]	0.197
Overall case fatality rate (%) in all NCUs	105/850	12.4	82/1049	7.8	0.001
Neonatal admissions that were preterm/LBW per NCU, median [IQR]	49	[12–58]	41	[32–55]	0.866
Neonatal deaths that were preterm/LBW per NCU, median [IQR]	8	[6-10]	5	[4-7]	0.397
Case fatality rate (%) among preterm/LBW in all NCUs	52/283	18.4	40/343	11.7	0.018

## Discussion

The ABC approach bundled evidence-based interventions to reduce preventable neonatal deaths during 18 months of intervention, and significant declines in neonatal mortality were recorded post-intervention.

Providers showed significant improvements in knowledge, skills and confidence in the use of QI methodology for the analysis of system issues using different QI tool and developing, testing, and implementing change ideas using PDSA [23]. Using these skills, NCU staff developed different QI change ideas aimed to bridge quality gaps identified in the health system generally related to the lack of enough and skilled health care providers, adherence to protocols, lack of information and involvement of mothers and caregivers in neonatal care, and poor communication among health care providers. Change ideas included use of evidence-based practices, such as the involvement of parents and other caregivers in neonatal care which has been demonstrated to improve outcomes especially in the understaffed NCUs [24, 25]. Notably, many of tested changes were in the nursing scope of work, which is not surprising given the critical role of nurses in neonatal care. The literature shows that limiting rotation of nurses improves the relationship between them and parents which increases chances to identify patients needs, build trust and increase parent’s involvement in care [26]. However, it is important to note that the overall staffing levels in the NCU were low, with one nurse per shift caring for an average of over 100 newborns each quarter which is a global challenge for providing specialized care for small and sick newborns [9].

Several QI projects developed and implemented by health facility NCU teams aimed to bridge quality gaps related to provider capacity. Lack of neonatal care trainings among NCU staff was challenging and other studies have found that clinical staff are appointed without formal trainings in newborn care [27]. ABC provided clinical trainings followed by regular facility-based clinical mentorship to improve knowledge and skills of NCU staff. In addition, QI change ideas to continuously address capacity gaps were reported by NCUs. We anticipate that these combined efforts improved provider skills, and thus contributed to improved neonatal care and outcomes as has been demonstrated by findings from other literature from similar settings [27, 28].

Availability of medicines and essential equipment and supplies was generally acceptable at baseline, with the exception of more advanced equipment for the care of small and sick newborns including syringe pumps, vital signs monitors, and CPAP. These gaps were directly addressed by ABC, however the challenges of maintaining available, functioning equipment and consumables was observed with some equipment such as radiant warmers decreasing over time. This persistent challenge of ensuring continuous availability of functioning equipment has been seen in other resource limited settings which shows gaps related to neonatal essential equipment availability and functionality that can hinder quality neonatal care [4, 29].

The combined ABC interventions led to a significant overall case fatality decline in seven NCUs in the period of 18 months. These findings from scale-up are comparable to those documented in the pilot implementation in two validation sites from 2013 to 2015 [19]. The notable decline in neonatal case fatality is not solely credited to the NCU QI interventions but to the overall implementation of the ABC bundle from the community health centers up to the district hospitals. A similar comprehensive intervention in rural Mozambique saw significant neonatal mortality reduction through improved infrastructure, provision of essential equipment, set up of protocols, improved organization, and trained clinical staff training [30]. The evidence-based practices to reduce neonatal mortality are available in the literature but translation into practice remains a challenge in low resource settings. The QI strategies designed by health facility teams in Rwanda show practical, feasible strategies for replication by facilities facing similar quality gaps.

Despite the significant improvements demonstrated by the ABC intervention, there are important challenges to note to inform further work in improving the quality of care for small and sick newborns. While newborn care is a national priority in Rwanda, a key challenge to the implementation of the project was the competing priorities within health facilities. It is essential to engage the health facility leadership in using QI by highlighting that it can be equally impactful on other district and health facility priorities. To address this, ABC QI Advisors occasionally coached the teams in services other than maternity and NCU to ensure the adoption of QI culture at the health facility level and made connections with the government’s priority initiative of health facility accreditation. In addition, the Memorandum of Understanding signed with the national MOH and district and hospital leaders was a key tool that aligned expectations for all stakeholders from the start of ABC. Additional challenges were related to staff turnover, insufficient number of staff, and insufficient functional ambulances for timely and safe referrals. While ABC was not able to address these challenges, advocacy to district and MOH leadership was made during the LCS and during national working groups of health stakeholders in Rwanda. These persistent challenges may highlight why birth asphyxia deaths did not decline significantly from baseline to endline. While ABC focused on essential skills such as improved intrapartum care and neonatal resuscitation, the causes of birth asphyxia are multifactorial [31]. The delayed transfers resulting from lack of sufficient ambulances, and non-optimal labor monitoring partly attributed to lack of sufficient staff play a role in the persistance of birth asphyxia cases.

## Limitations

This study relied on retrospectively reviewing the change ideas implemented, rather than documentation of change ideas and each PDSA cycle so we are unable to discern which change ideas resulted in the most change. In addition, measurement of improved QI skills relied on self-report assessment and not a direct examination of skills. The sample size is relatively small, with 7 hospital NCUs.

## Conclusions

ABC is an evidence-based bundle of interventions focused on improving the health system to have essential equipment, skilled staff, and a culture of data use and continuous improvement. QI change ideas targeted diverse quality challenges and were successfully implemented in facilities with limited NCU staffing. The ABC bundle is replicable at scale and contributed to significant declines in neonatal mortality in just 18 months.

## Data Availability

The datasets used and/or analyzed during the current study are available from the corresponding author on reasonable request.

## Abbreviations

ABC: All Babies Count
HMIS: Health Information System
KMC: Kangaroo Mother Care
LBW: Low Birthweight
LCS: Learning Collaborative Session
LMIC: Low- and Middle-Income Countries
MOH: Ministry of Health
NCU: Neonatal Care Unit
PIH/IMB: Partners In Health/Inshuti Mu Buzima
PDSA: Plan-Do-Study-Act
QI: Quality Improvement
